# Supplementary material for: Recombination and the role of pseudo-overdominance in polyploid evolution
Source: G3 (Bethesda). 2026 Feb 20;16(5):jkag048. doi: 10.1093/g3journal/jkag048 (PMC13148398; doi:10.1093/g3journal/jkag048)
Supplement: jkag048_Supplementary_Data [file jkag048_supplementary_data.pdf]

SUPPLEMENTAL INFORMATION

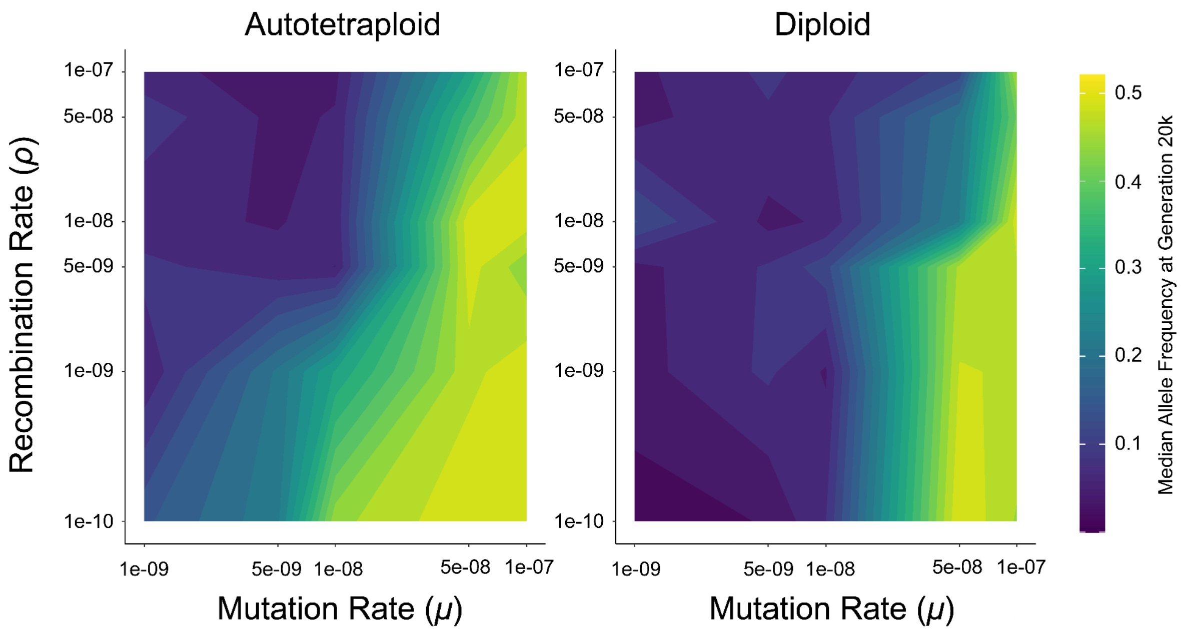

Supplemental Figure 1. Median allele frequency at 20,000 generations for autotetraploid (top) and diploid (bottom) simulations under the empirical *h*-*s* DFE model with a population of *N* = 100 individuals. Axes are transformed to log scale, with values between ticks interpolated to fill in the gradient. Allele frequencies for each parameter combination are averaged across 10 replicates.

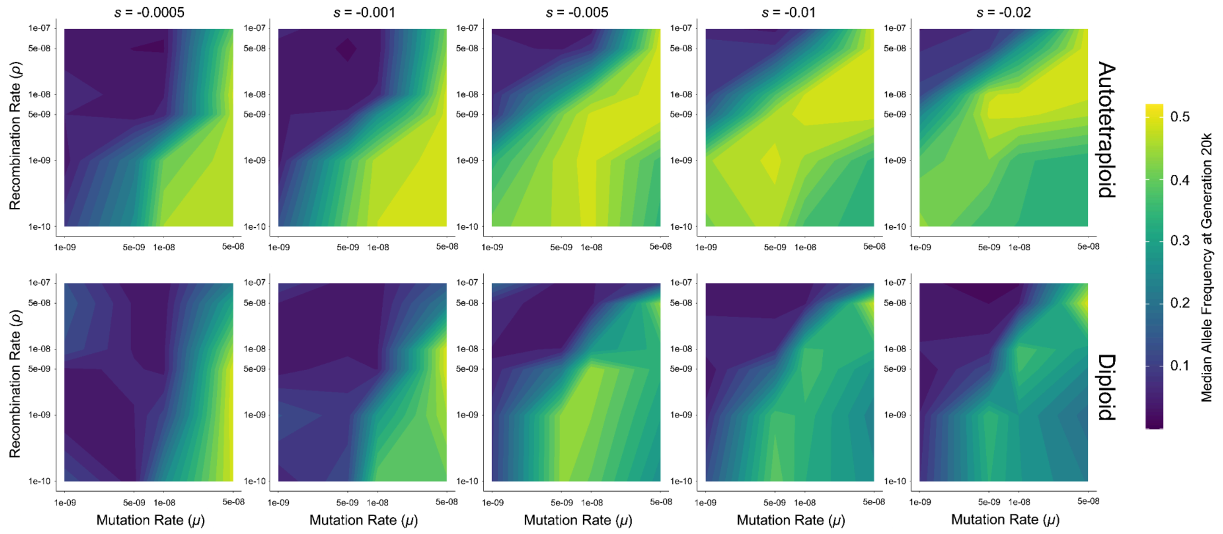

Supplemental Figure 2. Median allele frequency at 20,000 generations for autotetraploid (top) and diploid (bottom) simulations of a population of *N* = 200 individuals. Axes are transformed to log scale, with values between ticks interpolated to

fill in the gradient. Allele frequencies for each parameter combination are averaged across 10 replicates. Note, mutation rate at 1e-07 missing at this *N* as simulations were unable to finish due to the high mutation rate accumulating many mutations at low recombination rates.

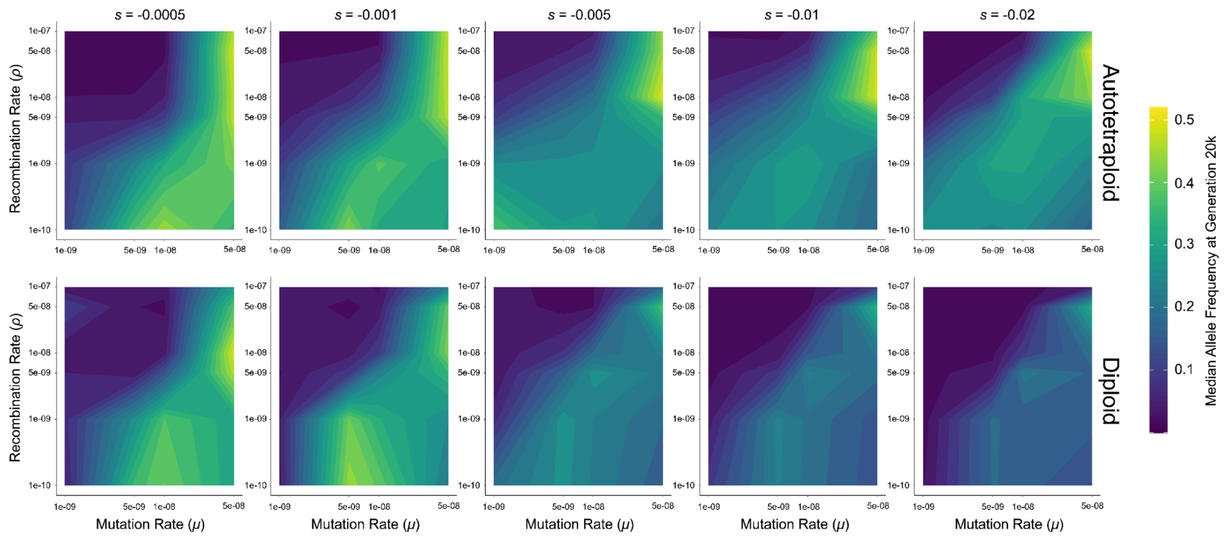

Supplemental Figure 3. Median allele frequency at 20,000 generations for autotetraploid (top) and diploid (bottom) simulations of a population of *N* = 500 individuals. Axes are transformed to log scale, with values between ticks interpolated to fill in the gradient. Allele frequencies for each parameter combination are averaged across 10 replicates. Note, mutation rate at 1e-07 missing at this *N* as simulations were unable to finish due to the high mutation rate accumulating many mutations at low recombination rates.

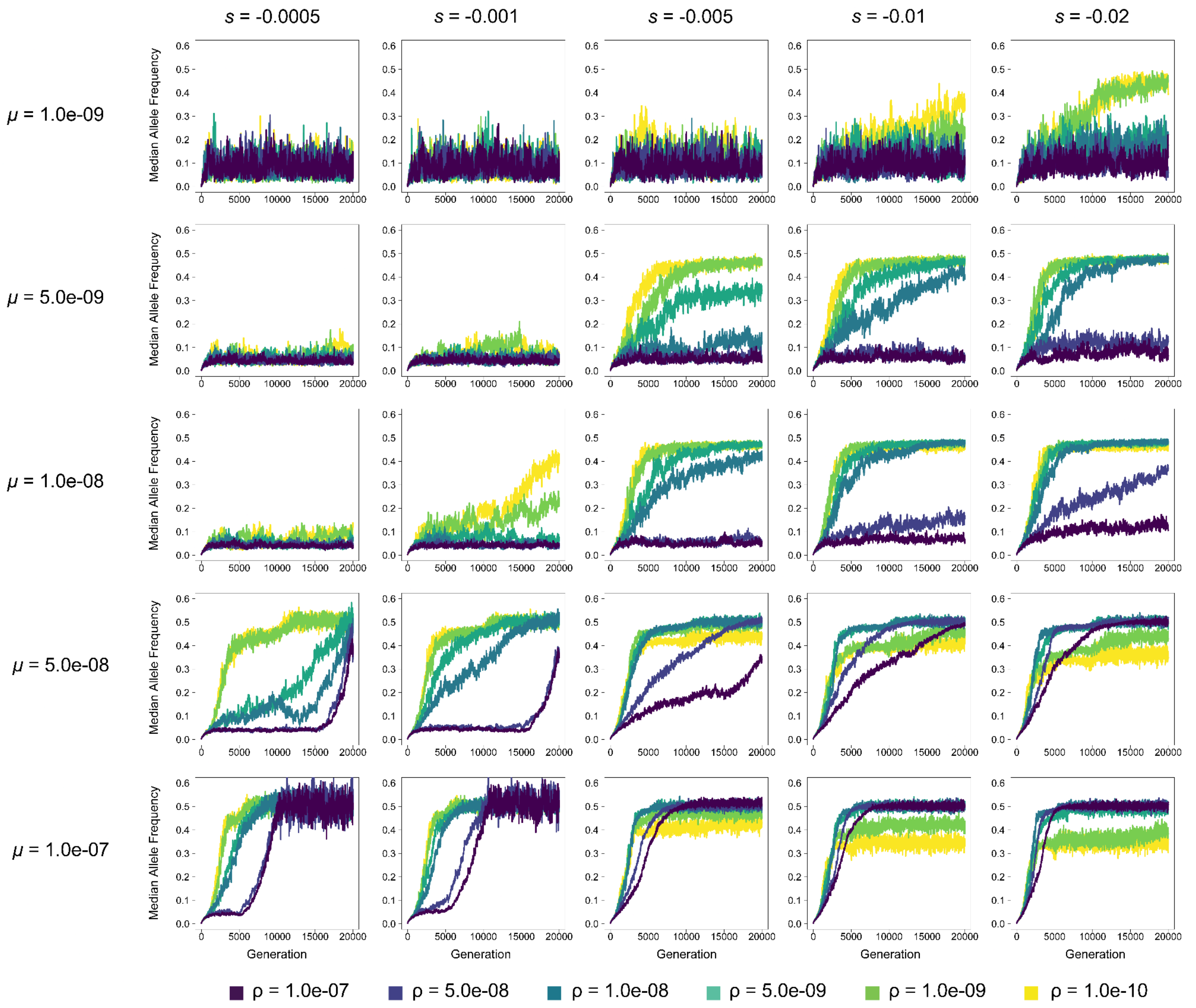

Supplemental Figure 4. Median allele frequency of autotetraploids in each generation at varying mutation rates (μ) and selection coefficients (*s*) for *N* = 100. Each line represents the average across 10 replicates. Note: y-axis is identical across subfigures.

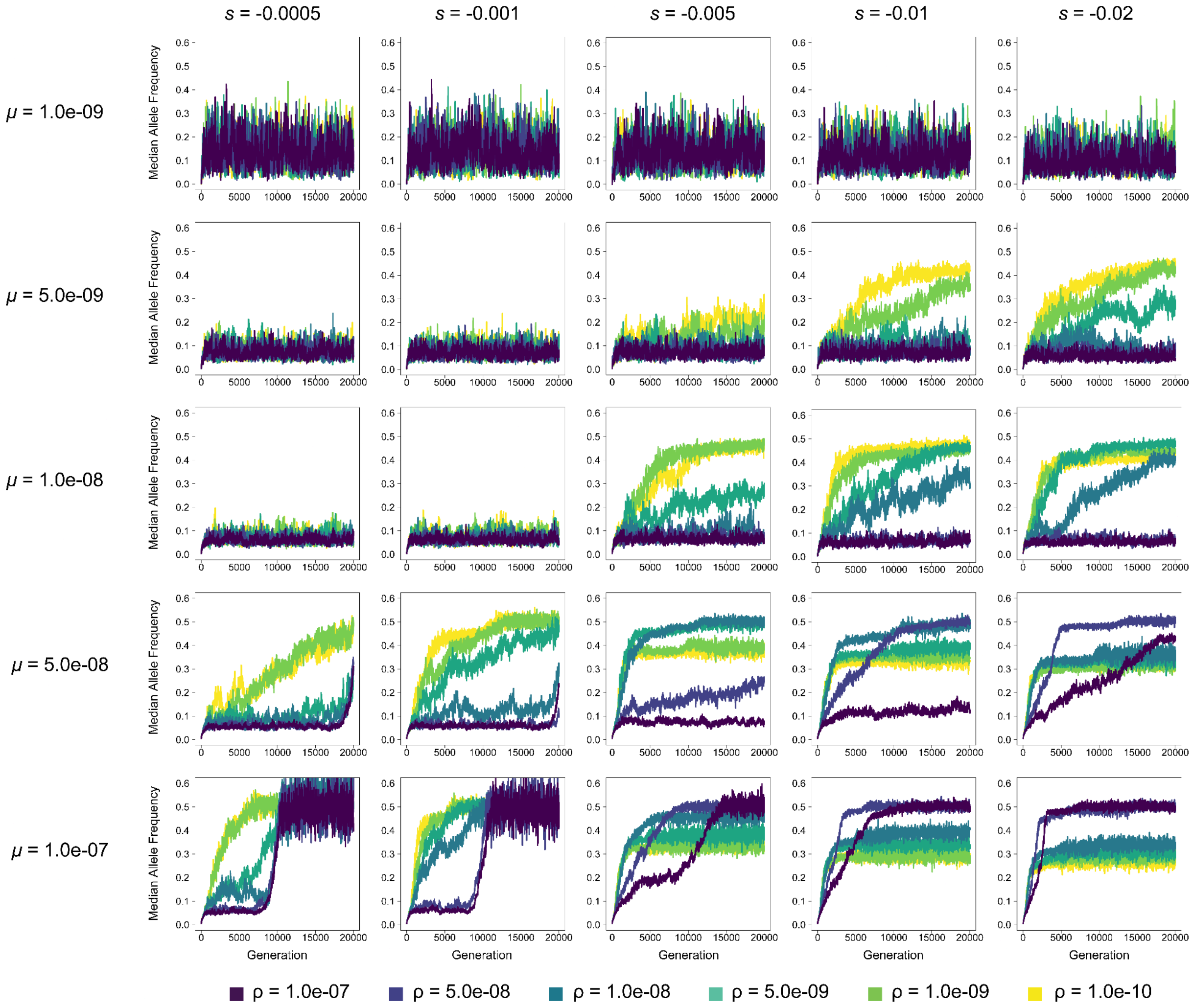

Supplemental Figure 5. Median allele frequency of diploids in each generation at varying mutation rates (μ) and selection coefficients (*s*) for *N* = 100. Each line represents the average across 10 replicates. Note: y-axis is identical across subfigures.

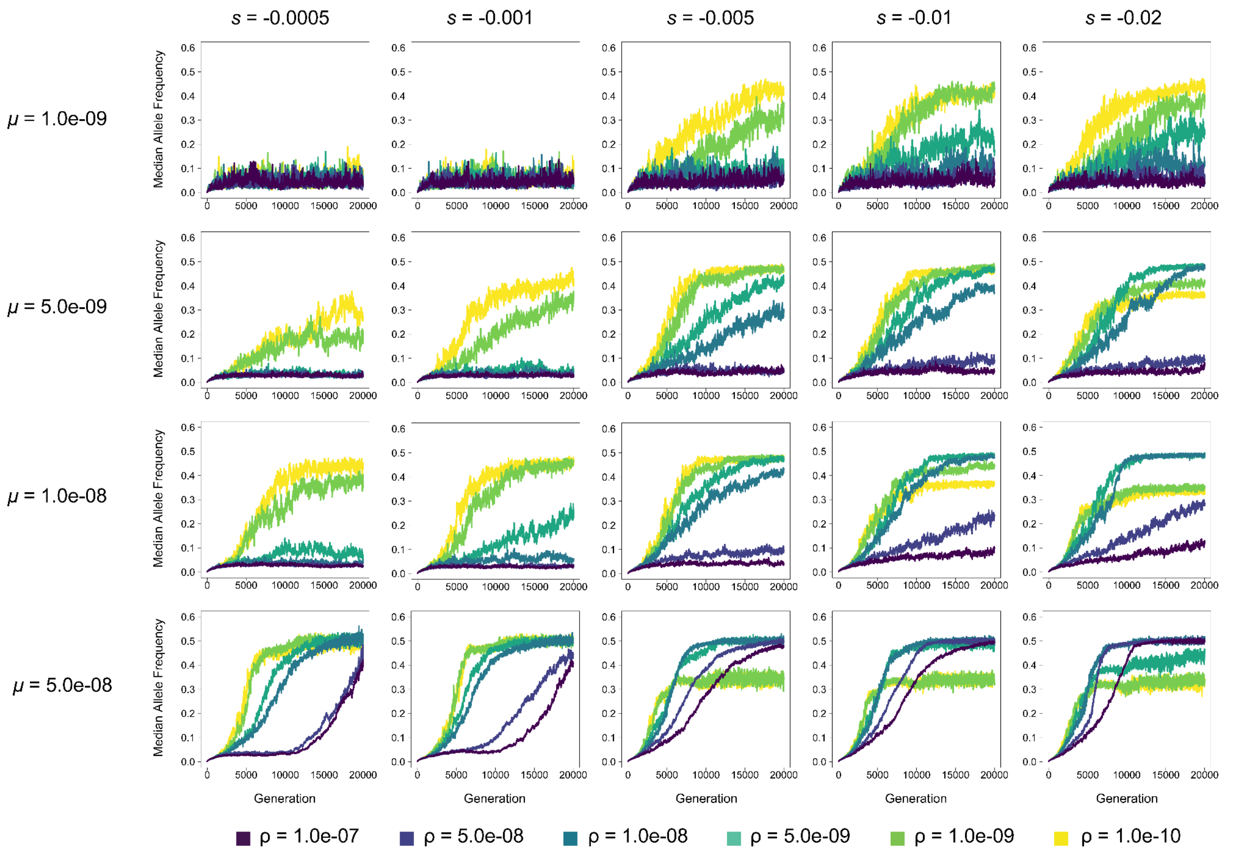

Supplemental Figure 6. Median allele frequency of autotetraploids in each generation at varying mutation rates (μ) and selection coefficients (*s*) for *N* = 200. Each line represents the average across 10 replicates. Note: y-axis is identical across subfigures, and that μ=1e-7 simulations are not included here because these replicates could not complete in the maximum allotted time on our compute cluster for all *N*.

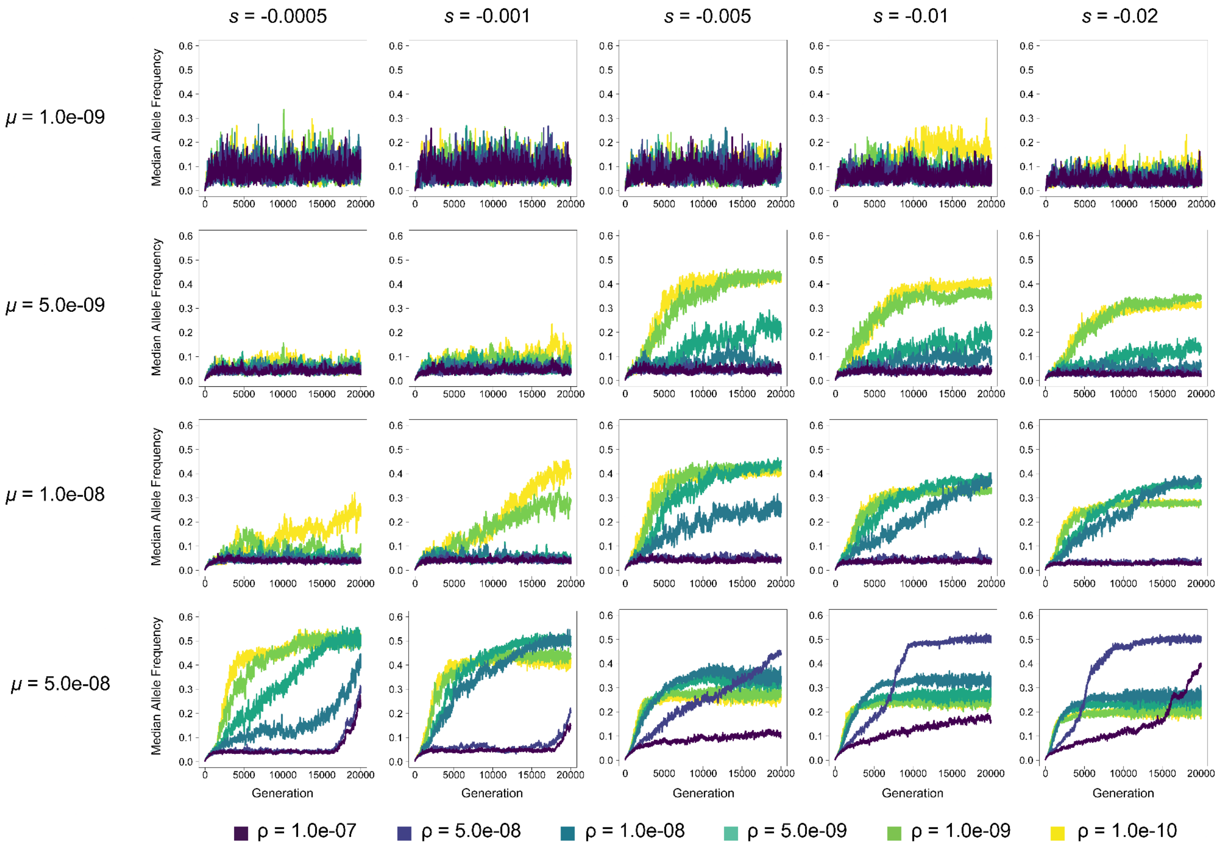

Supplemental Figure 7. Median allele frequency of diploids in each generation at varying mutation rates (μ) and selection coefficients (s) for N = 200. Each line represents the average across 10 replicates. Note: y-axis is identical across subfigures.

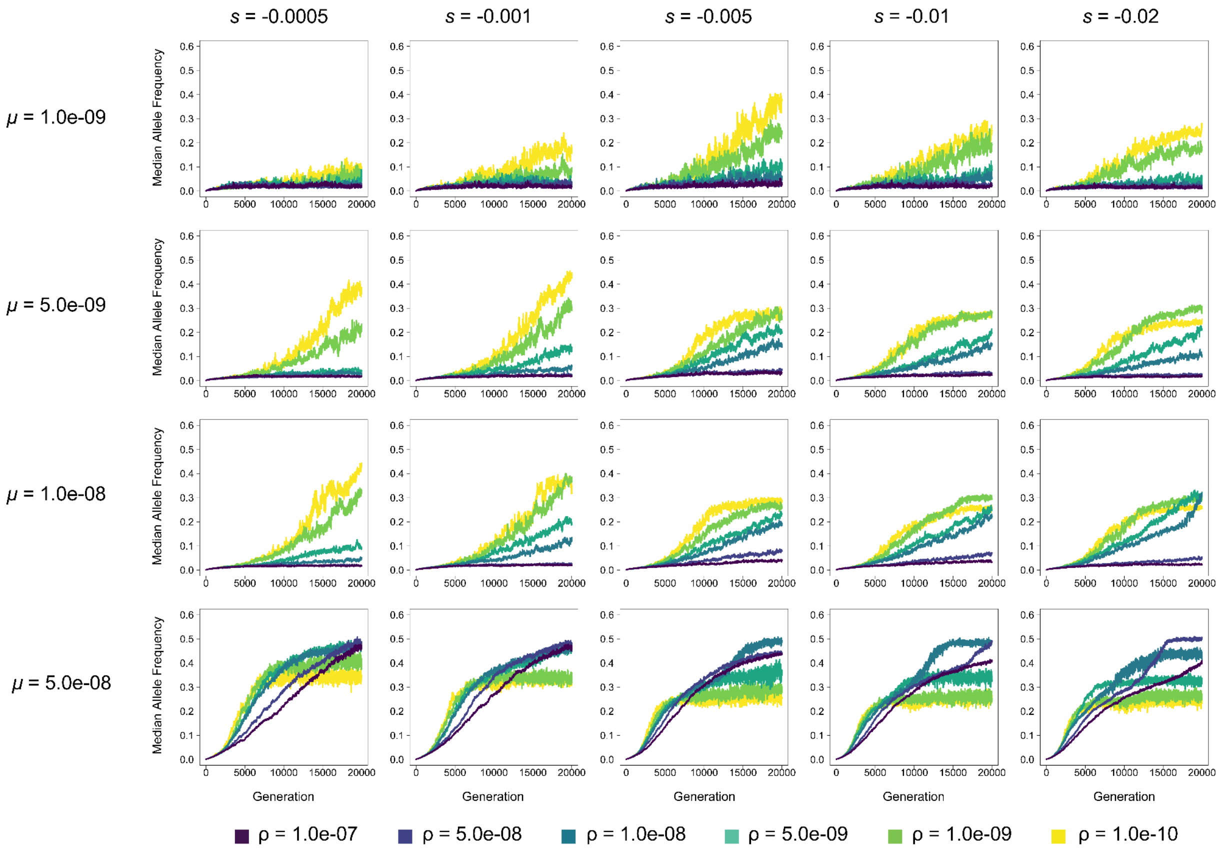

Supplemental Figure 8. Median allele frequency of autotetraploids in each generation at varying mutation rates (μ) and selection coefficients (*s*) for *N* = 500. Each line represents the average across 10 replicates. Note: y-axis is identical across subfigures.

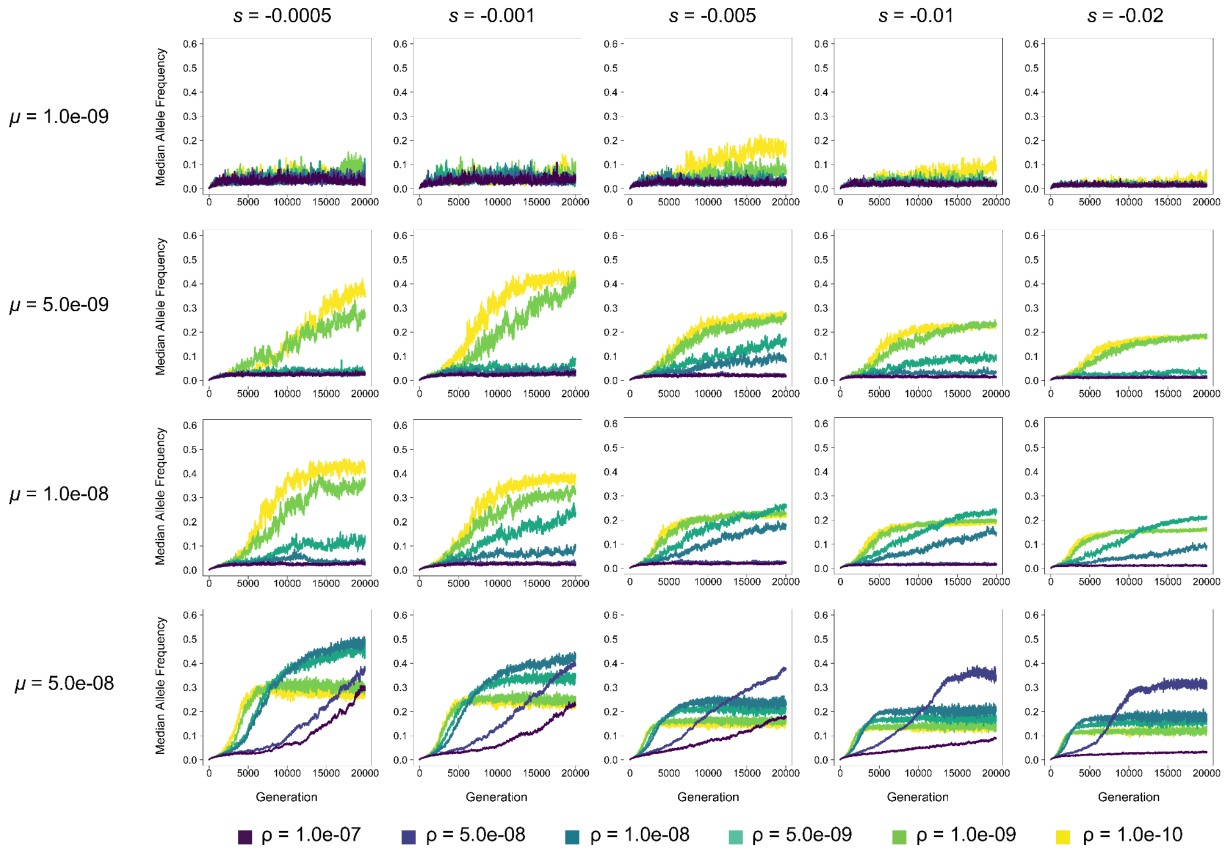

Supplemental Figure 9. Median allele frequency of diploids in each generation at varying mutation rates (μ) and selection coefficients (s) for N = 500. Each line represents the average across 10 replicates. Note: y-axis is identical across subfigures.

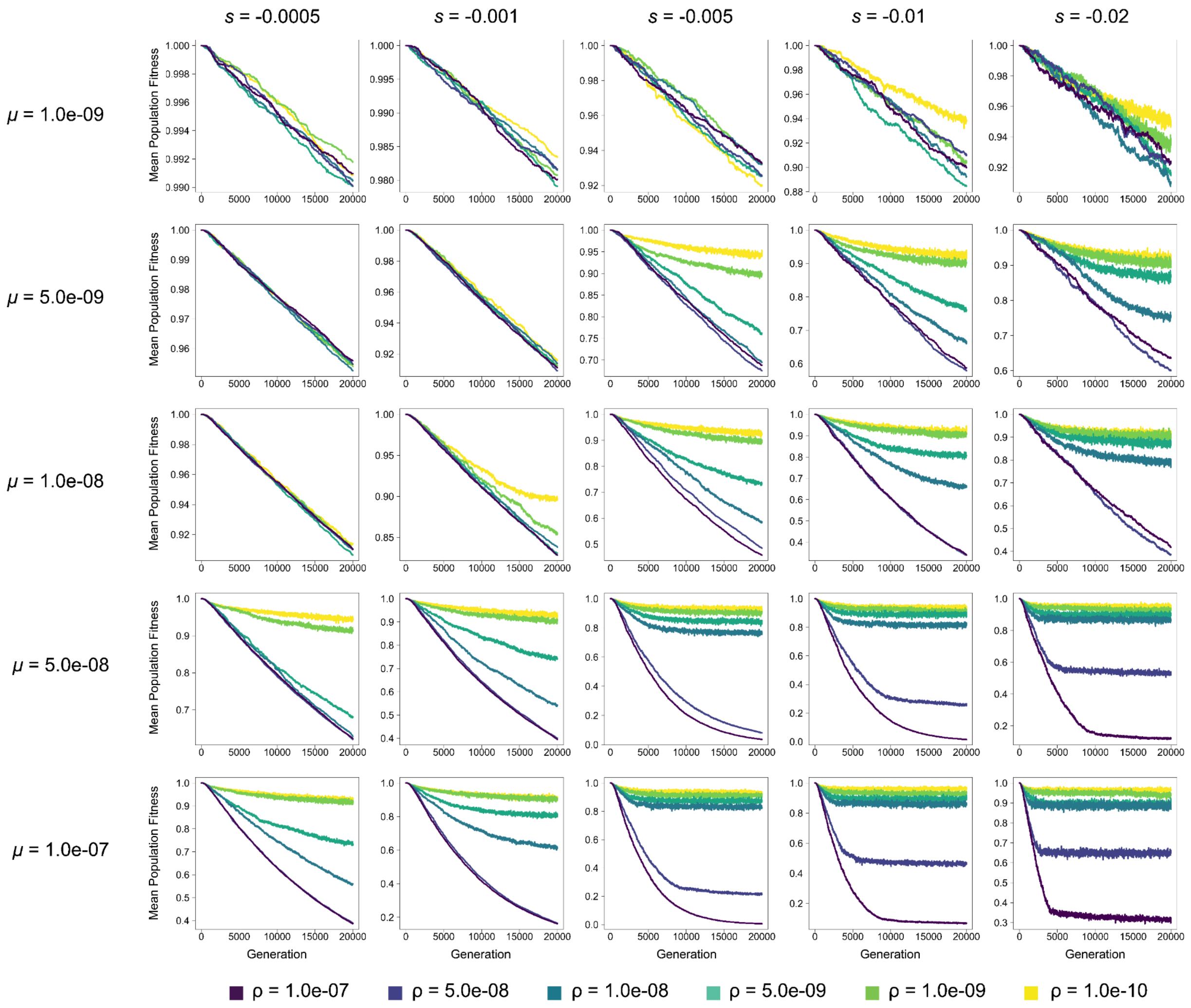

Supplemental Figure 10. Mean population fitness of autotetraploids in each generation at varying mutation rates (μ) and selection coefficients (s) for N = 100. Each line represents the average across 10 replicates. Note: y-axis varies across subfigures.

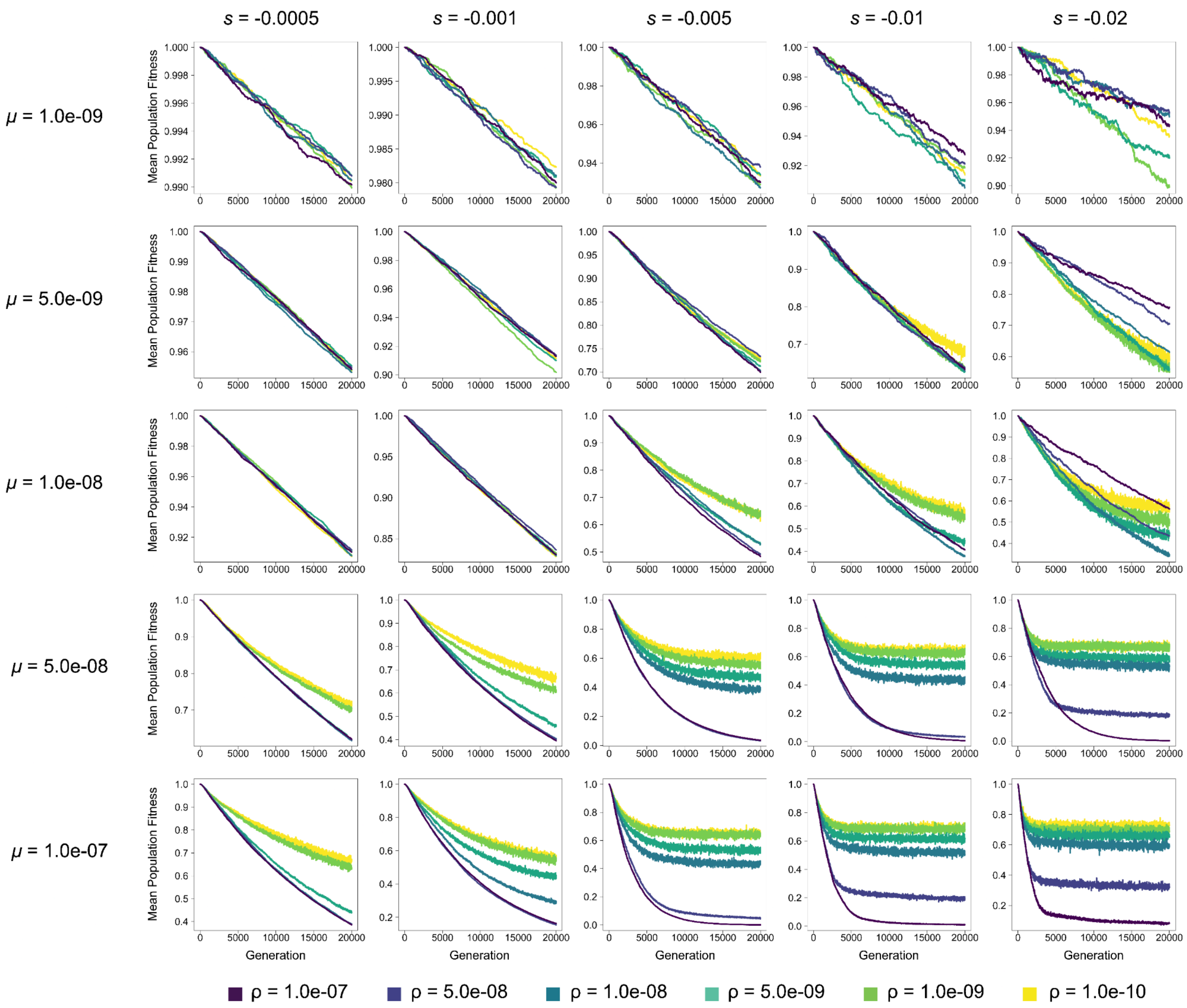

Supplemental Figure 11. Mean population fitness of diploids in each generation at varying mutation rates (μ) and selection coefficients (s) for N = 100. Each line represents the average across 10 replicates. Note: y-axis varies across subfigures.

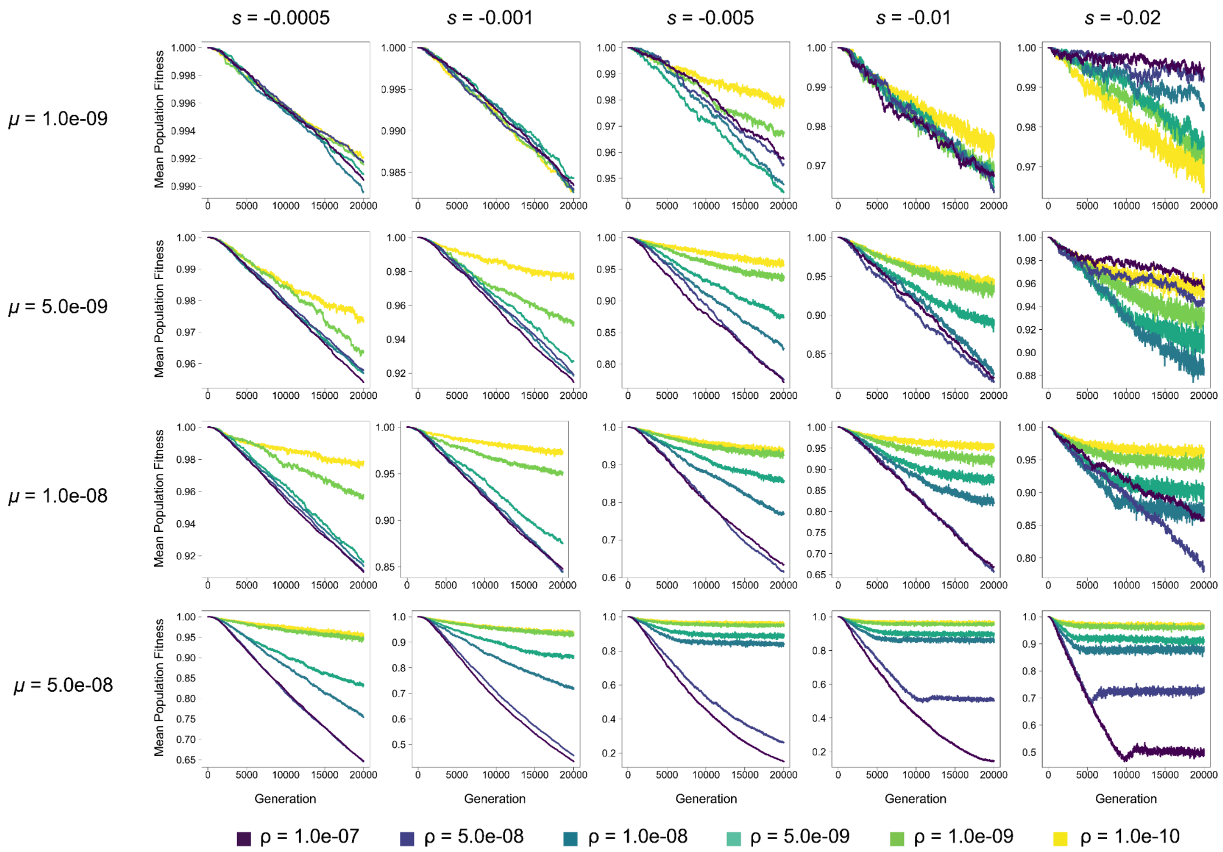

Supplemental Figure 12. Mean population fitness of autopolyploids in each generation at varying mutation rates (μ) and selection coefficients (*s*) for *N* = 200. Each line represents the average across 10 replicates. Note: y-axis varies across subfigures.

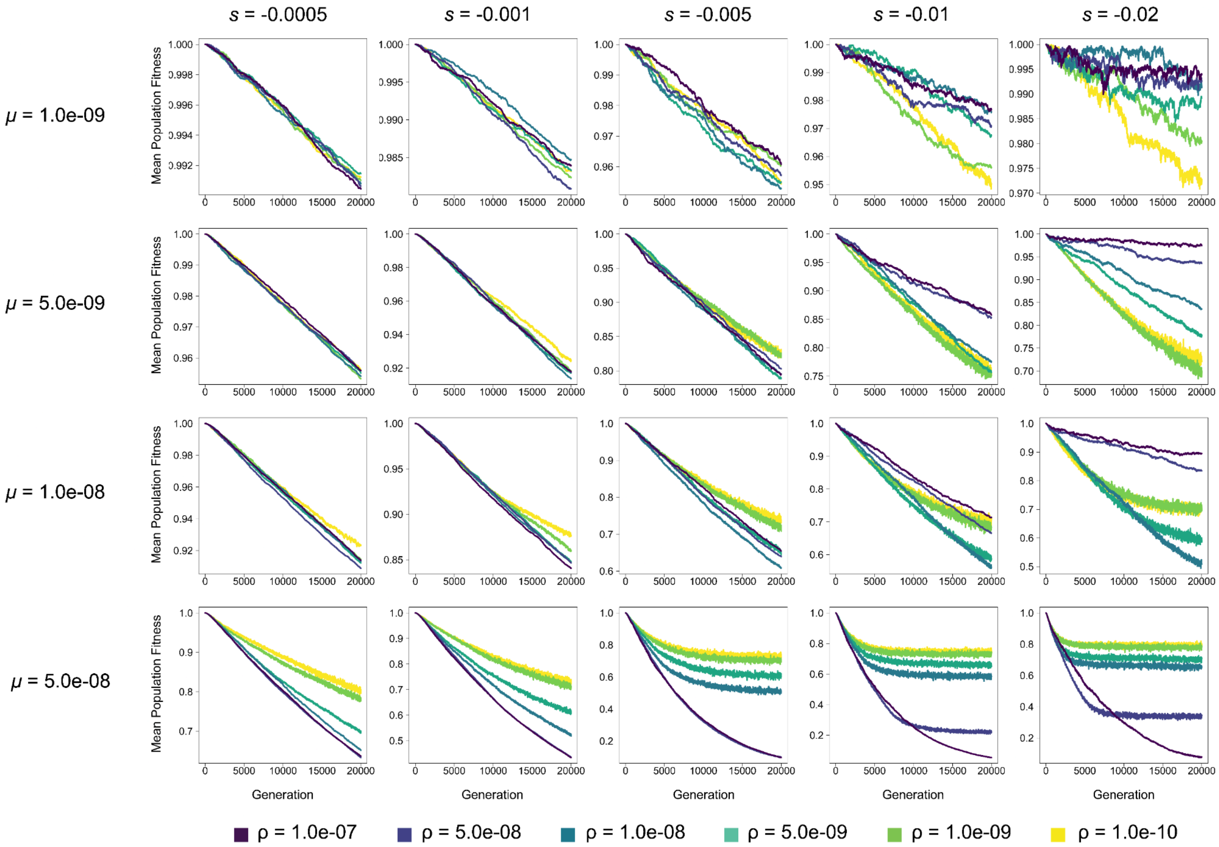

Supplemental Figure 13. Mean population fitness of diploids in each generation at varying mutation rates (μ) and selection coefficients (s) for N = 200. Each line represents the average across 10 replicates. Note: y-axis varies across subfigures.

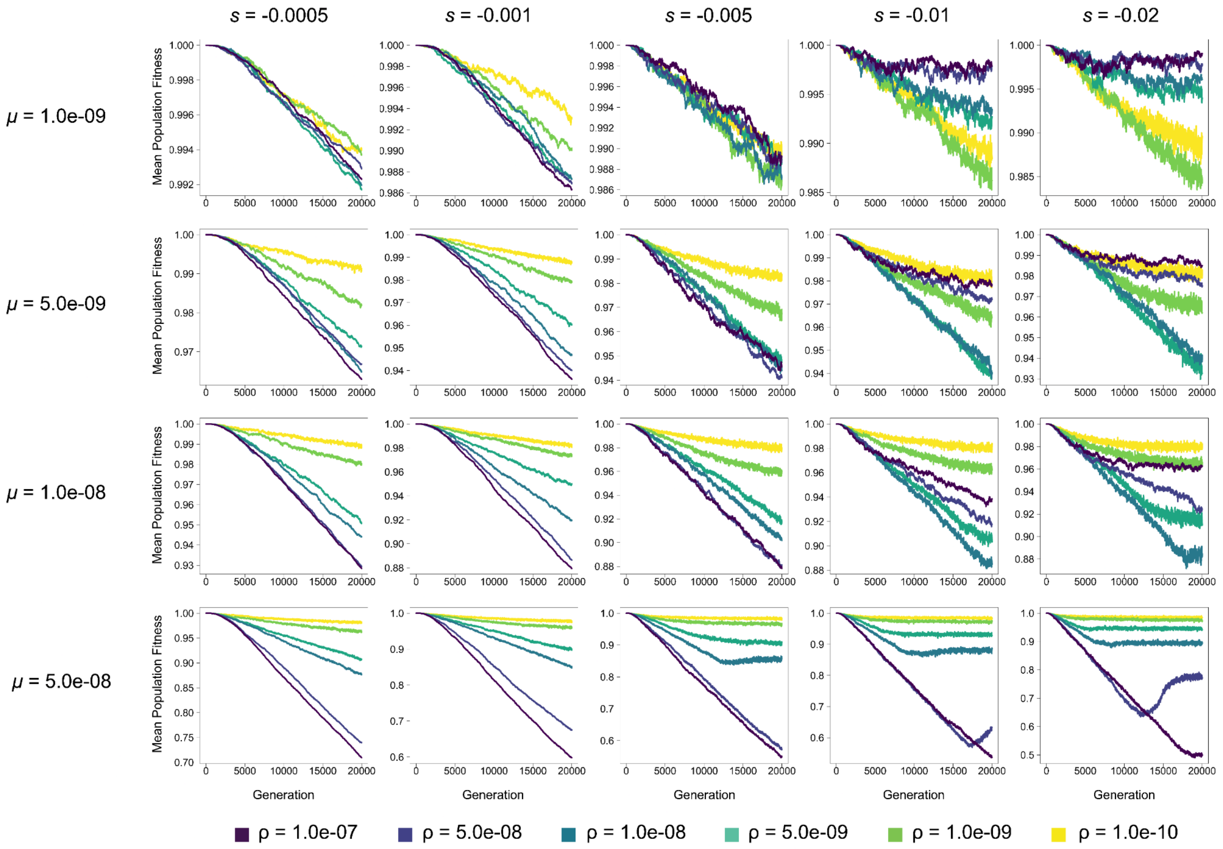

Supplemental Figure 14. Mean population fitness of autopolyploids in each generation at varying mutation rates (μ) and selection coefficients (s) for N = 500. Each line represents the average across 10 replicates. Note: y-axis varies across subfigures.

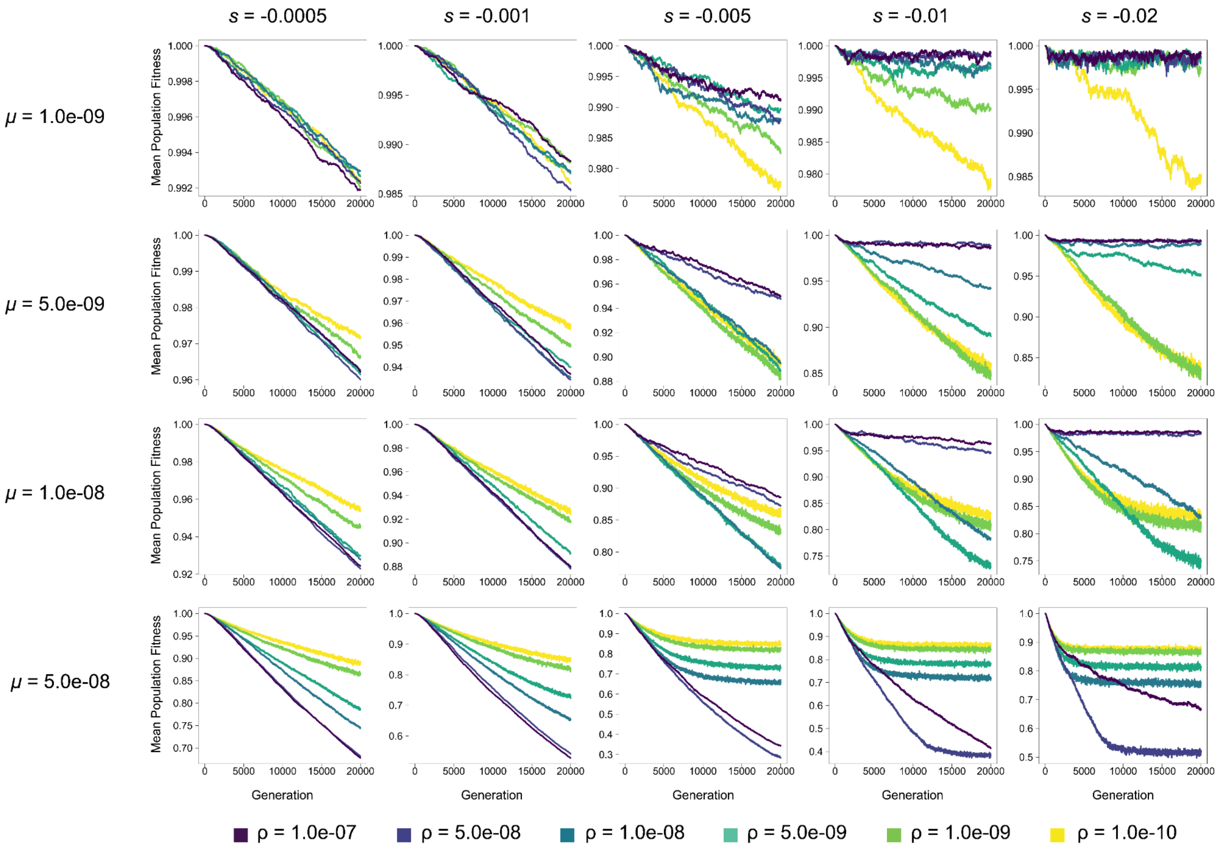

Supplemental Figure 15. Mean population fitness of diploids in each generation at varying mutation rates (μ) and selection coefficients (*s*) for *N* = 500. Each line represents the average across 10 replicates. Note: y-axis varies across subfigures.

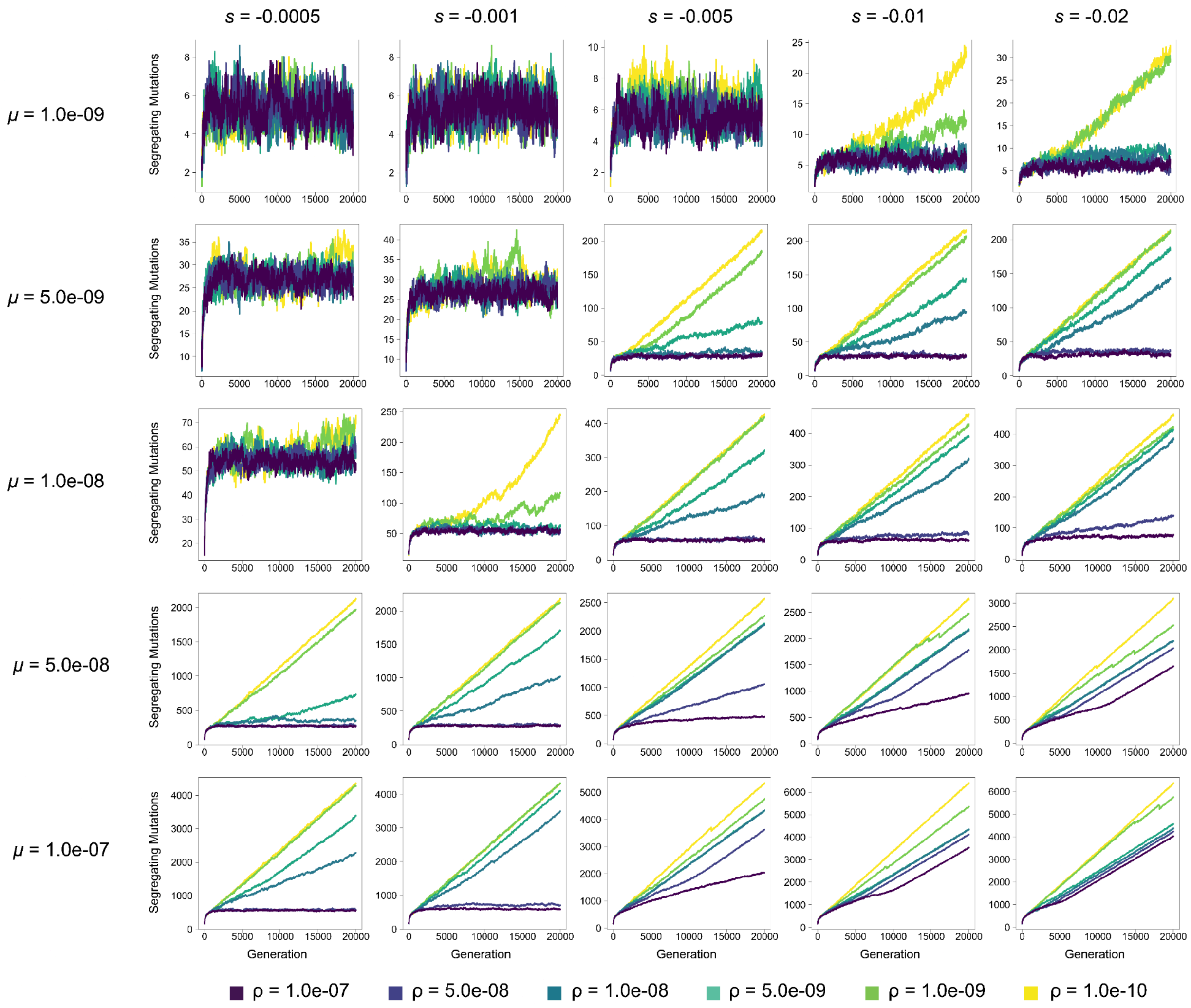

Supplemental Figure 16. The number of segregating mutations in autotetraploid populations in each generation at varying mutation rates (μ) and selection coefficients (*s*) for *N* = 100. Each line represents the average across 10 replicates. Note: y-axis varies across subfigures.

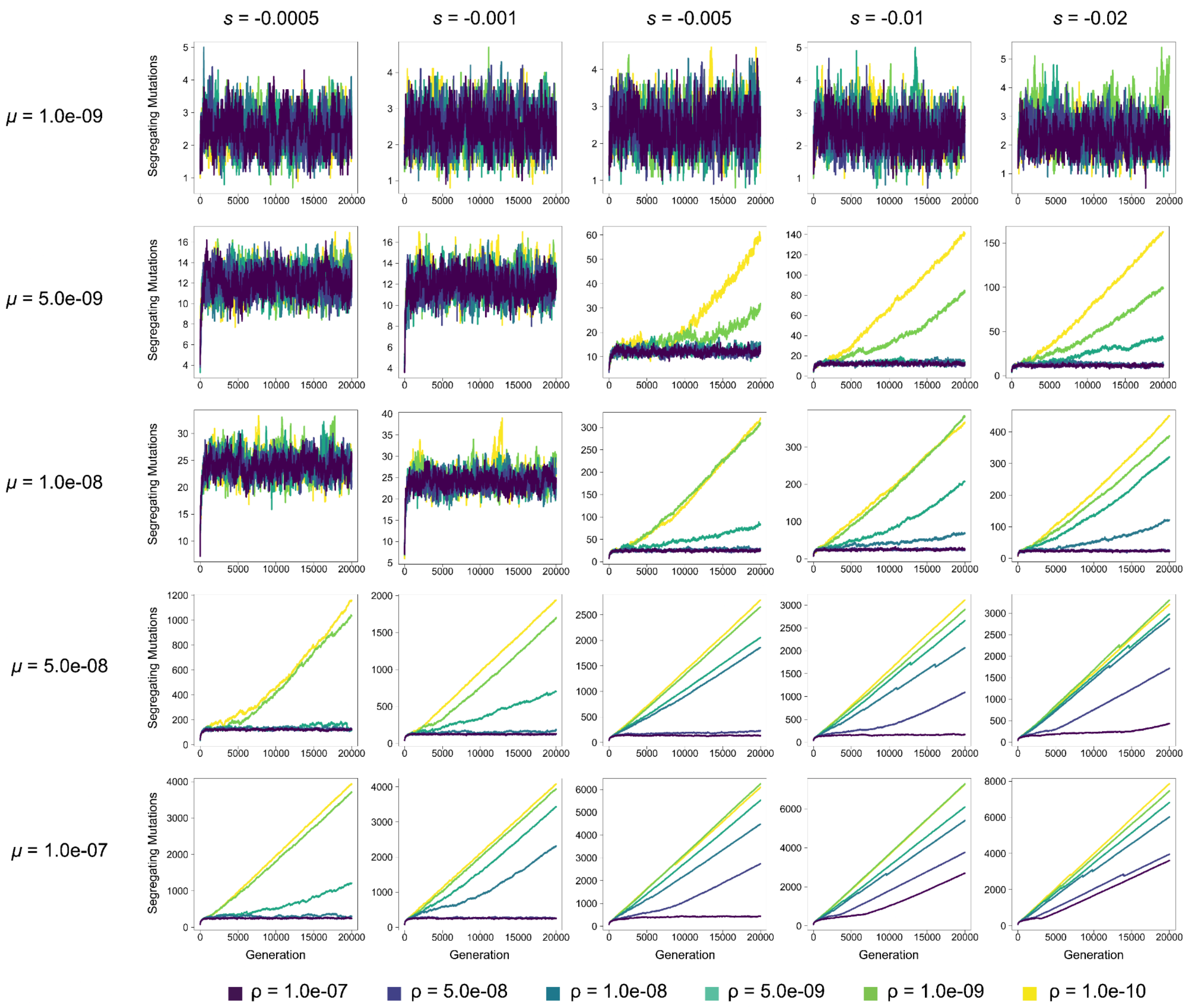

Supplemental Figure 17. The number of segregating mutations in diploid populations in each generation at varying mutation rates (μ) and selection coefficients (s) for N = 100. Each line represents the average across 10 replicates. Note: y-axis varies across subfigures.

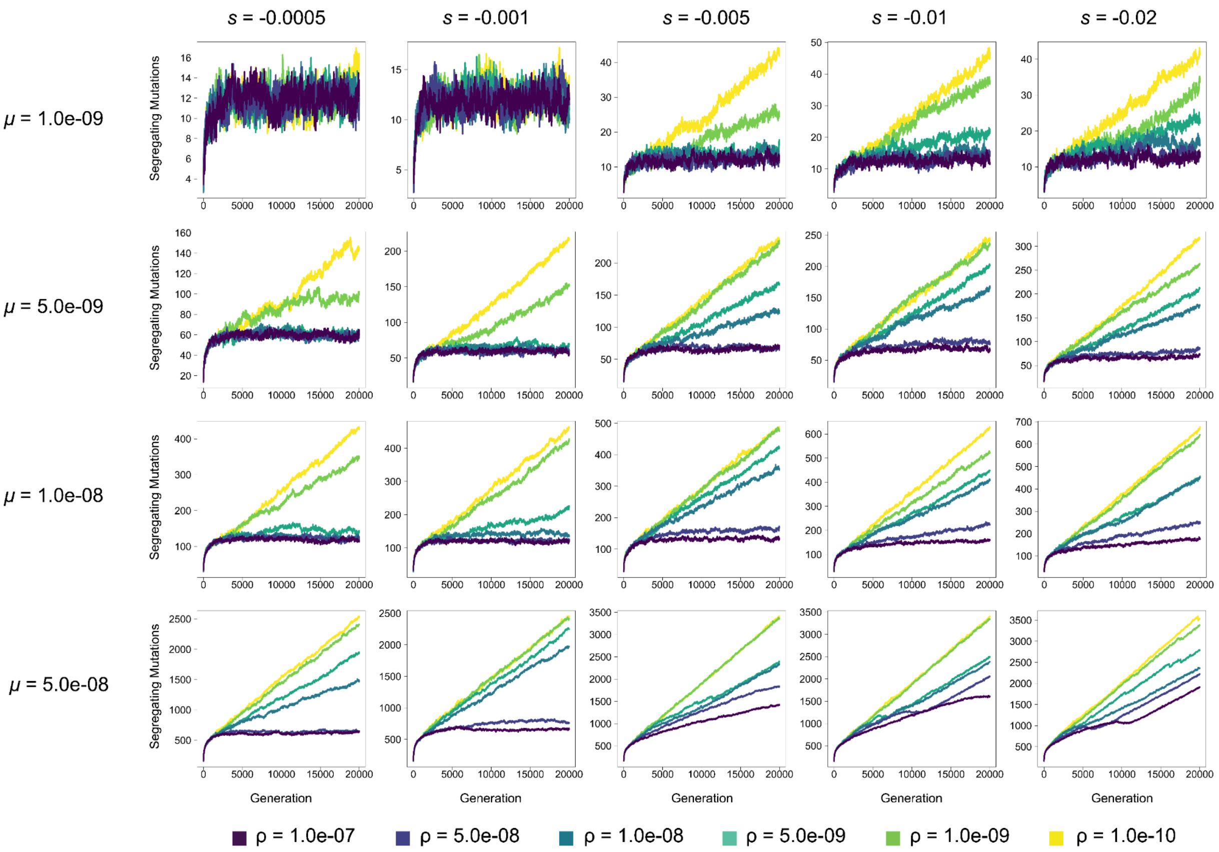

Supplemental Figure 18. The number of segregating mutations in autotetraploid populations in each generation at varying mutation rates (μ) and selection coefficients (s) for N = 200. Each line represents the average across 10 replicates. Note: y-axis varies across subfigures.

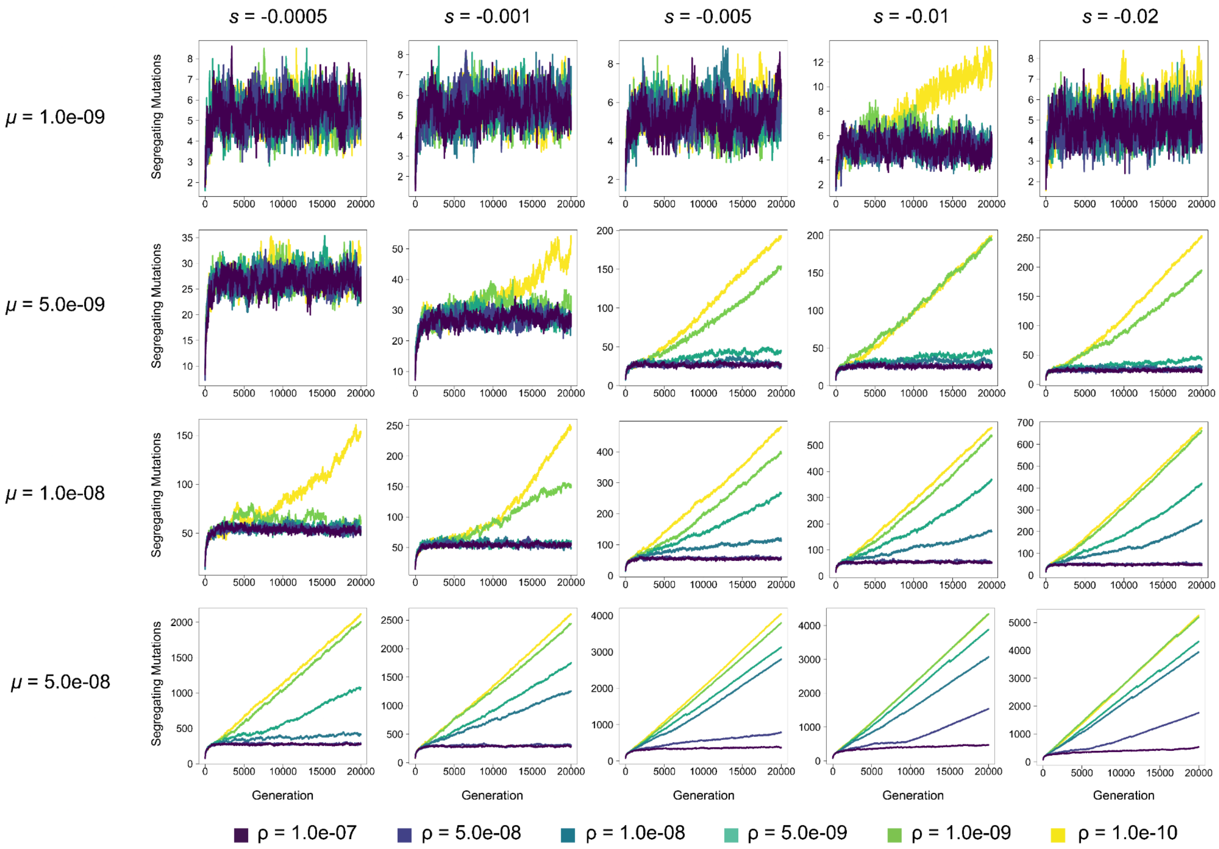

Supplemental Figure 19. The number of segregating mutations in diploid populations in each generation at varying mutation rates (μ) and selection coefficients (*s*) for *N* = 200. Each line represents the average across 10 replicates. Note: y-axis varies across subfigures.

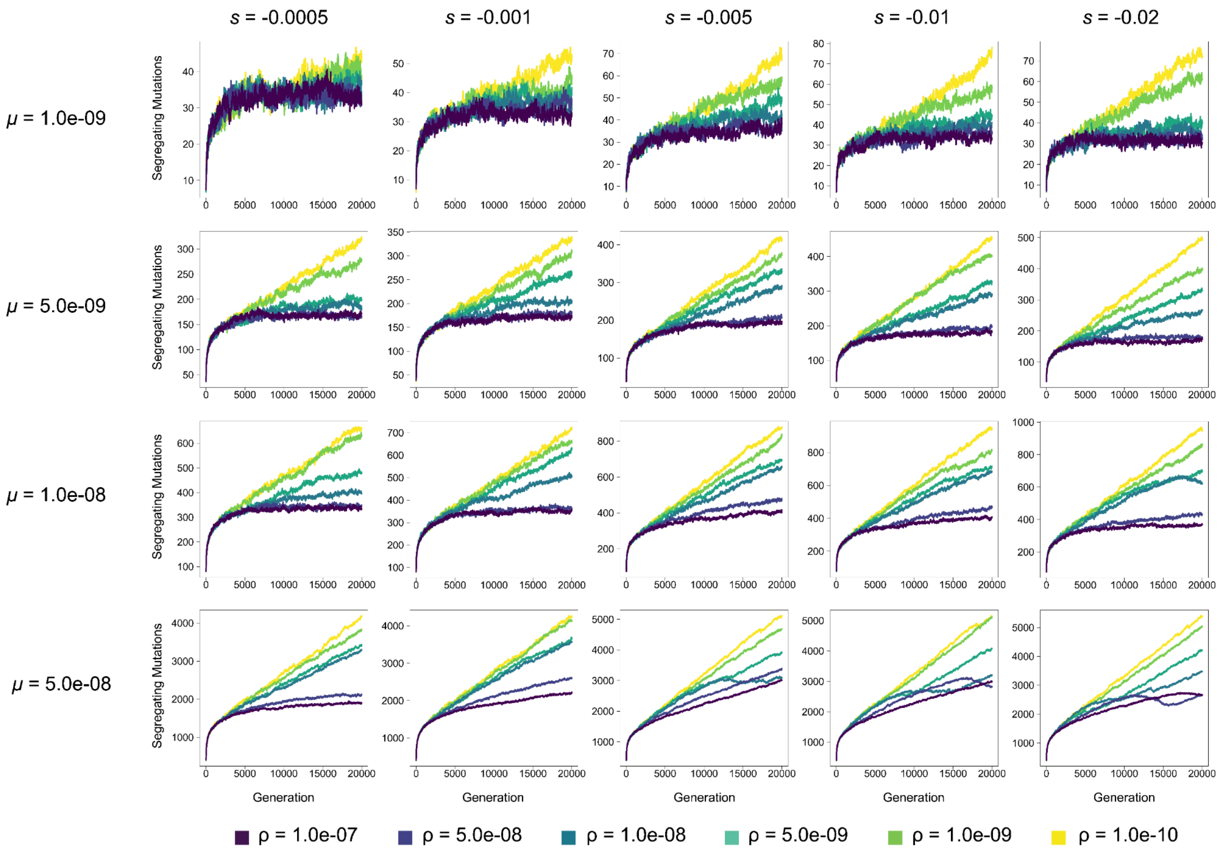

Supplemental Figure 20. The number of segregating mutations in autotetraploid populations in each generation at varying mutation rates (μ) and selection coefficients (*s*) for *N* = 500. Each line represents the average across 10 replicates. Note: y-axis varies across subfigures.

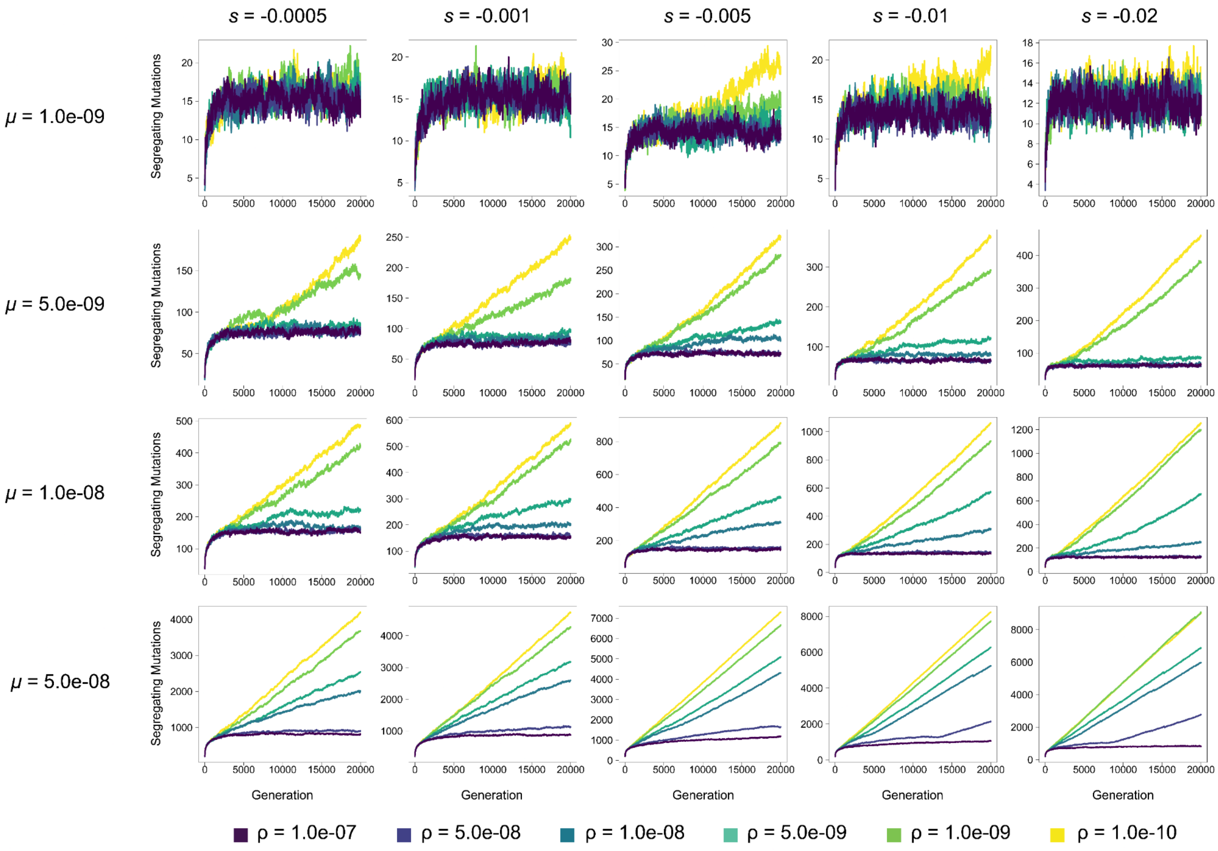

Supplemental Figure 21. The number of segregating mutations in diploid populations in each generation at varying mutation rates (μ) and selection coefficients (s) for N = 500. Each line represents the average across 10 replicates. Note: y-axis varies across subfigures.

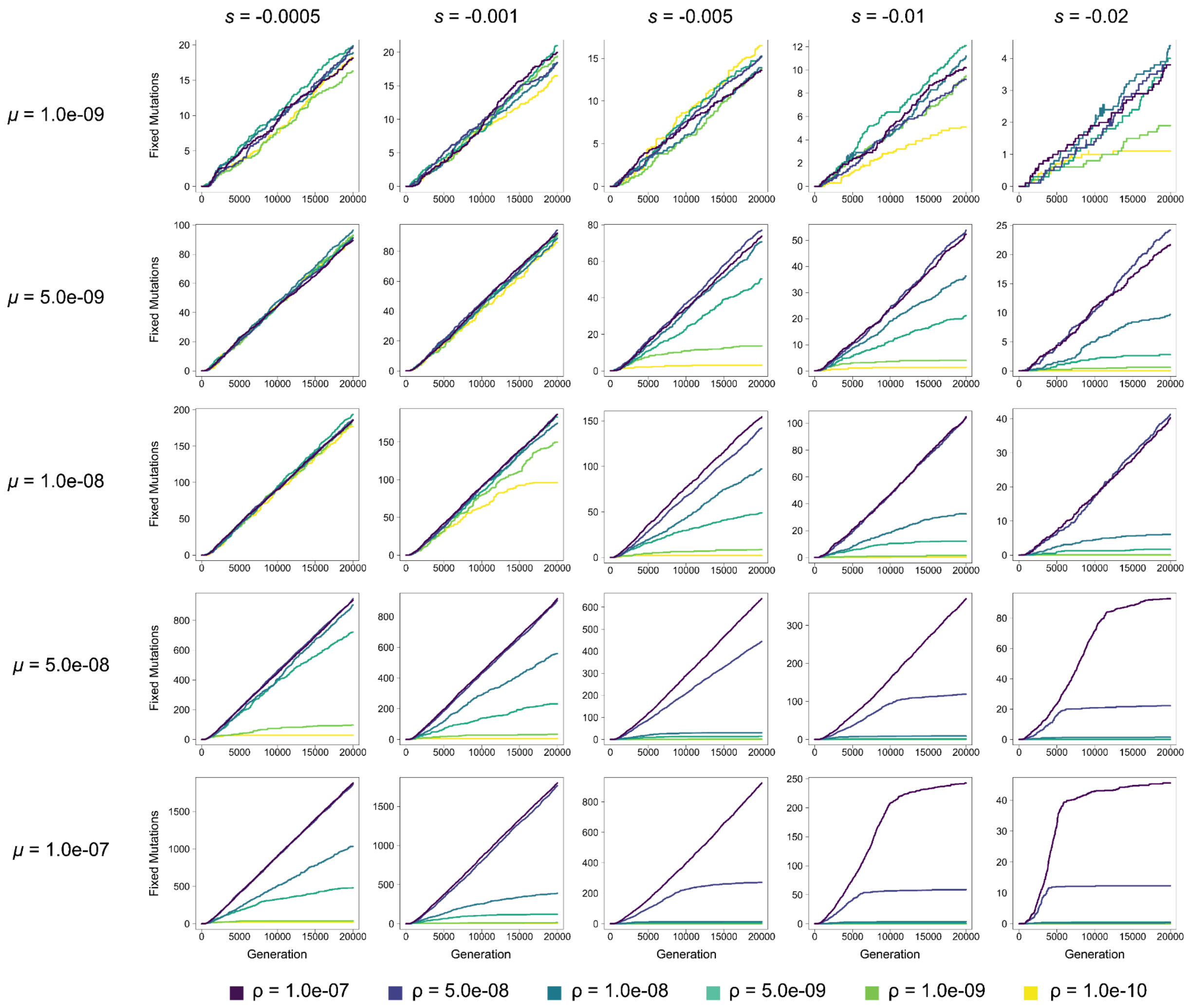

Supplemental Figure 22. The number of fixed mutations in autotetraploid populations in each generation at varying mutation rates (μ) and selection coefficients (s) for N = 100. Each line represents the average across 10 replicates. Note: y-axis varies across subfigures.

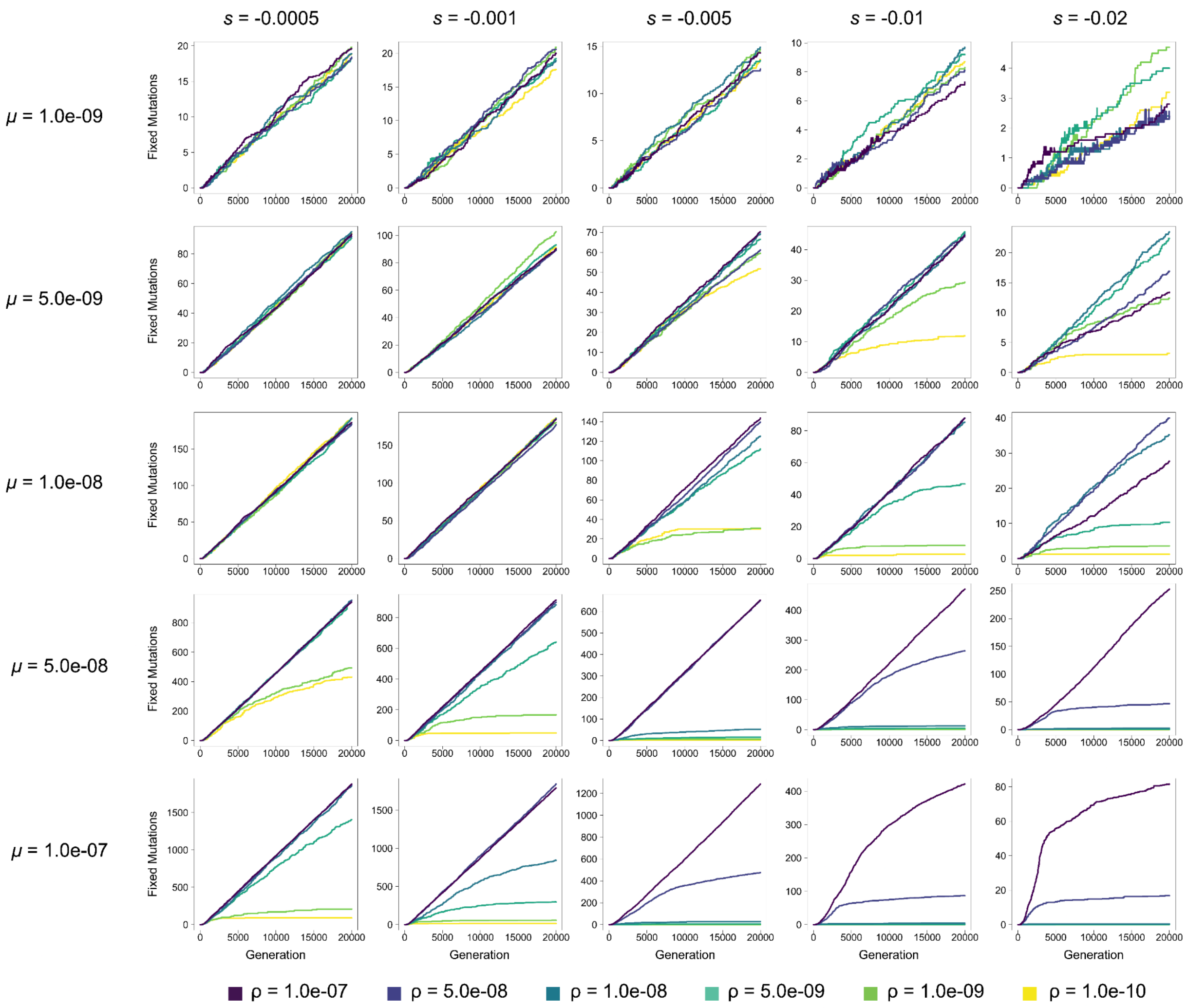

Supplemental Figure 23. The number of fixed mutations in diploid populations in each generation at varying mutation rates (μ) and selection coefficients (*s*) for *N* = 100. Each line represents the average across 10 replicates. Note: y-axis varies across subfigures.

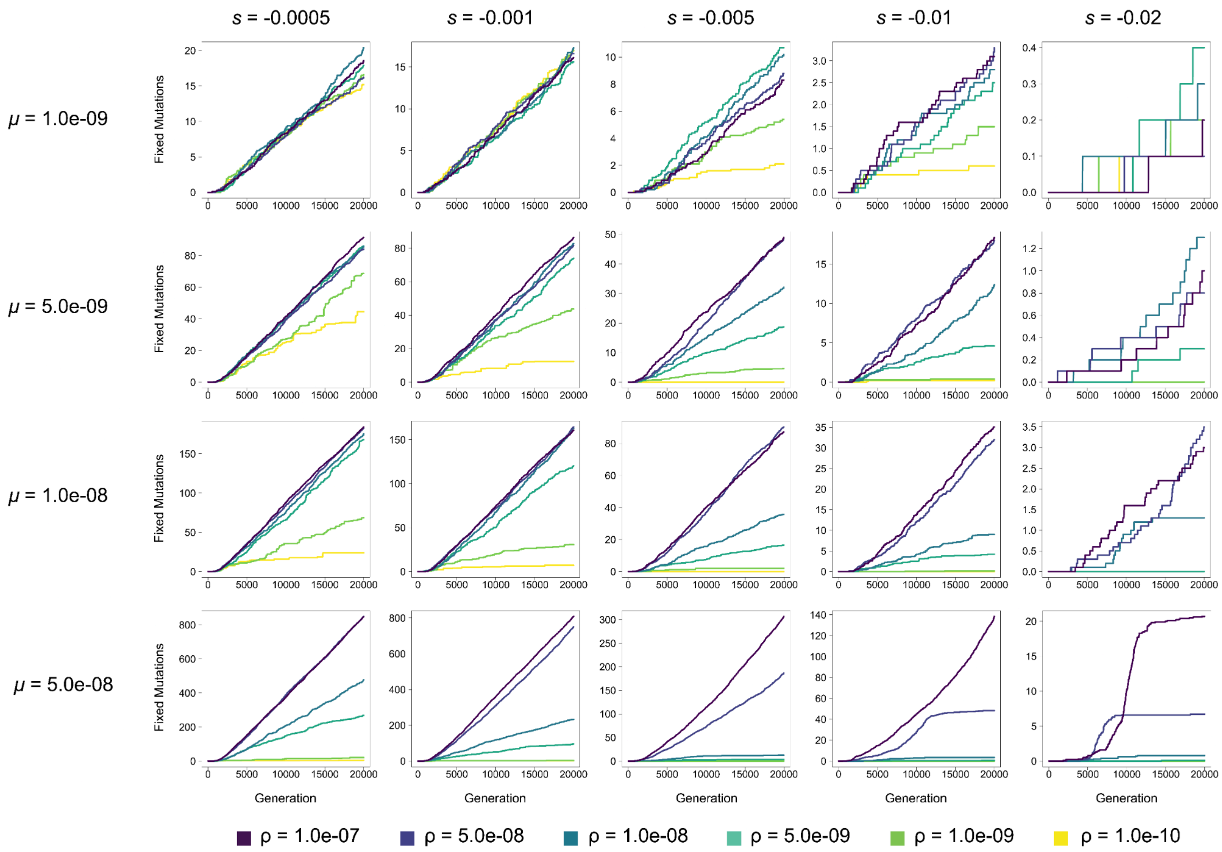

Supplemental Figure 24. The number of fixed mutations in autotetraploid populations in each generation at varying mutation rates (μ) and selection coefficients (s) for N = 200. Each line represents the average across 10 replicates. Note: y-axis varies across subfigures.

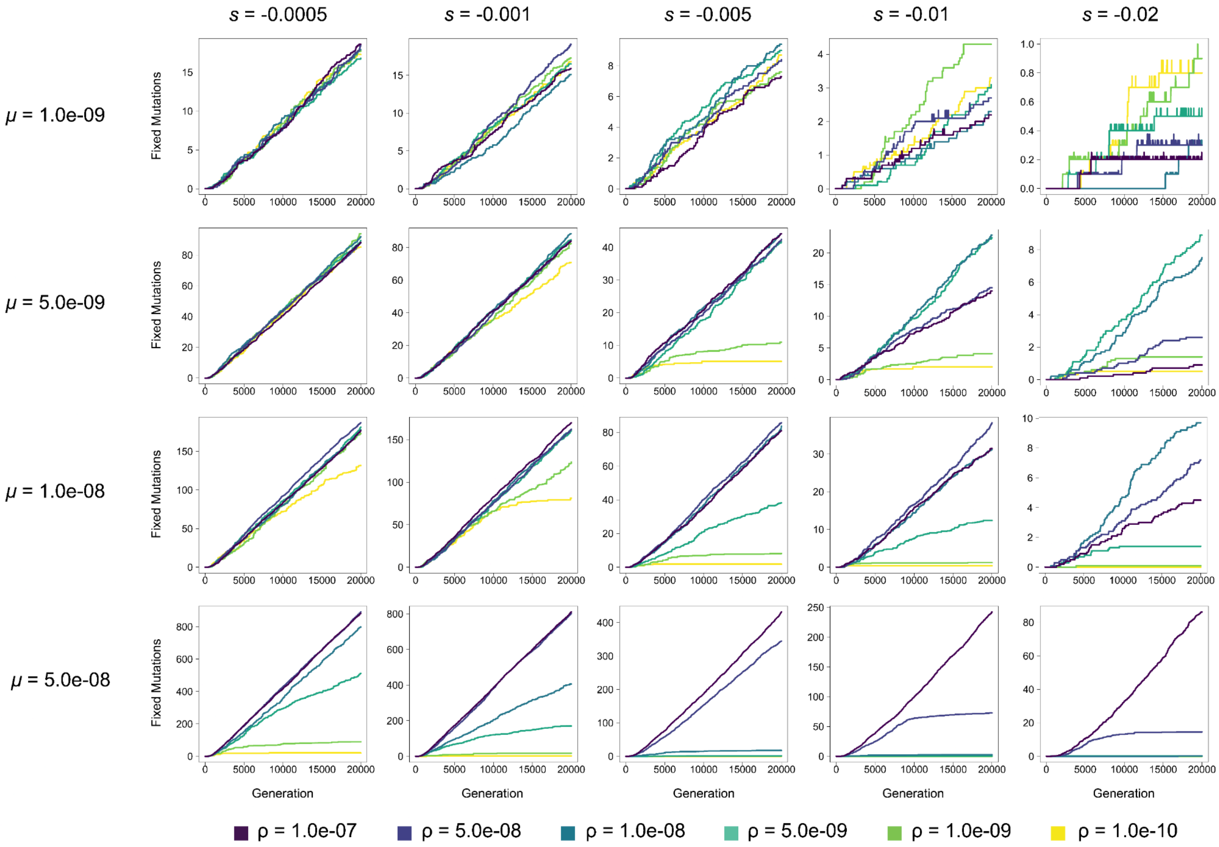

Supplemental Figure 25. The number of fixed mutations in diploid populations in each generation at varying mutation rates (μ) and selection coefficients (*s*) for *N* = 200. Each line represents the average across 10 replicates. Note: y-axis varies across subfigures.

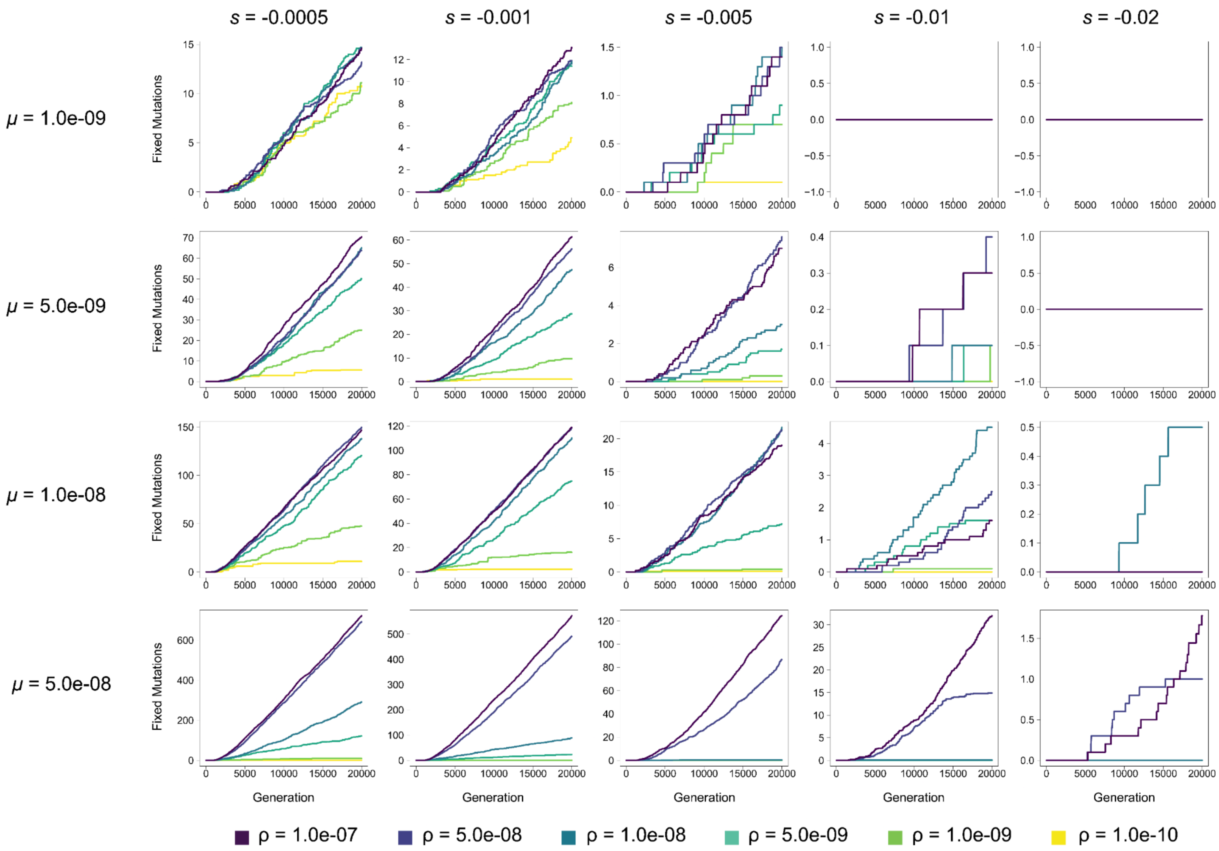

Supplemental Figure 26. The number of fixed mutations in autotetraploid populations in each generation at varying mutation rates (μ) and selection coefficients (*s*) for *N* = 500. Each line represents the average across 10 replicates. Note: y-axis varies across subfigures.

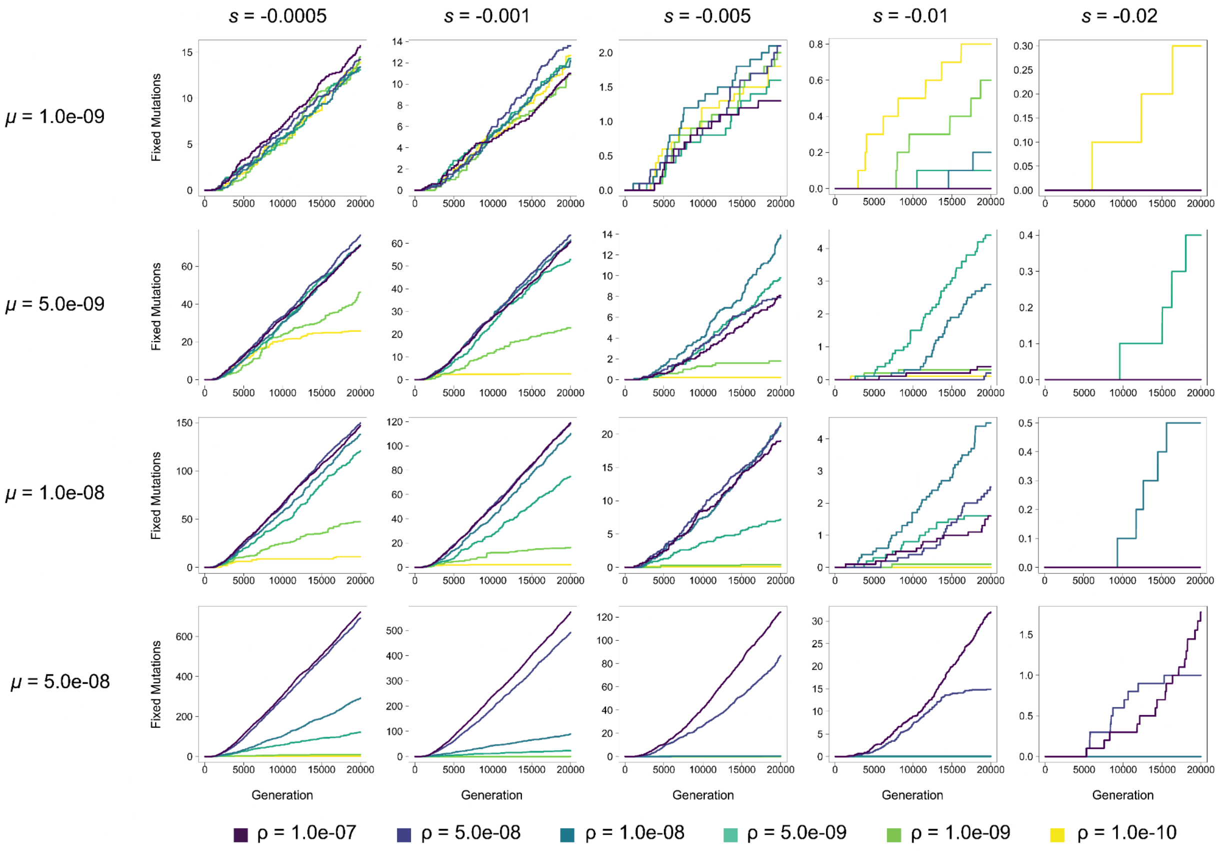

Supplemental Figure 27. The number of fixed mutations in diploid populations in each generation at varying mutation rates (μ) and selection coefficients (s) for N = 500. Each line represents the average across 10 replicates. Note: y-axis varies across subfigures.
